# Systemic inflammatory regulators and preeclampsia: a two-sample bidirectional Mendelian randomization study

**DOI:** 10.3389/fgene.2024.1359579

**Published:** 2024-03-22

**Authors:** Chu Li, Yishu Tian, Djouhayna Dougarem, Litao Sun, Zixing Zhong

**Affiliations:** ^1^ Center for Reproductive Medicine, Department of Obstetrics, Zhejiang Provincial People’s Hospital (Affiliated People’s Hospital), Hangzhou Medical College, Hangzhou, China; ^2^ Center for Reproductive Medicine, Department of Ultrasound Medicine, Zhejiang Provincial People’s Hospital (Affiliated People’s Hospital), Hangzhou Medical College, Hangzhou, China; ^3^ Hospital of Obstetrics and Gynecology, Zhejiang University School of Medicine, Hangzhou, China

**Keywords:** cytokines, inflammation, preeclampsia, pregnancy, Mendelian randomization study

## Abstract

**Background:**

Systemic inflammatory regulators have been associated with preeclampsia (PE) during pregnancy; however, there is inconsistent evidence from animal models and observational results.

**Methods:**

Using summary data from genome-wide association studies (GWASs), we performed a bidirectional Mendelian randomization (MR) analysis of two samples of systemic inflammatory regulators (*n* = 8,186) and PE (*n* = 267,242) individuals of European ancestry. As our primary analysis, we used the random-effects inverse-variance weighted (IVW) approach. Sensitivity and pleiotropy analyses were conducted using the MR–Egger method, weighted median, MR Pleiotropy RESidual Sum and Outlier (MR-PRESSO), and Cochran’s Q test.

**Results:**

The results indicate that there is a correlation between a higher circulating level of tumor necrosis factor alpha (TNF-α) and interleukin-9 (IL-9) and an increased risk of PE (odds ratio [OR] = 1.32, 95% confidence interval [CI] = 1.09–1.60, *p* = 0.004 and OR = 1.28, 95% CI: 1.02–1.62, *p* = 0.033, respectively). Conversely, lower levels of stem cell growth factor beta (SCGF-β) (OR = 0.89, 95% CI: 0.80–0.99, *p* = 0.027) and interleukin-5 (IL-5) (OR = 0.80, 95% CI: 0.65–0.98, *p* = 0.030) are linked to an increased risk of PE. The macrophage migration inhibitory factor (MIF) is the downstream inflammatory regulator of PE, according to reverse magnetic resonance imaging studies.

**Conclusion:**

Our study suggests that SCGF-β, IL-5, IL-9, and TNF-α causally affect the PE risk, while PE is causally associated with MIF. Further studies are needed to validate these biomarkers in managing PE.

## Introduction

### Background

Preeclampsia (PE) is a common and severe pregnancy complication, which is one of the leading causes of maternal mortality worldwide (Ives et al., 2020). It is characterized by new-onset hypertension after 20 weeks of gestation and multiple organ dysfunction (Brown et al., 2018). Women who survive PE may have a shorter life expectancy and an increased risk of stroke, cardiovascular disease, and diabetes (Poon et al., 2019; Kvalvik et al., 2020; Pittara et al., 2021). However, the mechanism of this hypertensive disorder in pregnancy remains elusive. Increasing evidence shows that the balance between pro- and anti-inflammatory factors is an intrinsic mechanism of occurrence and development of PE.

Several studies have indicated the potential involvement of systemic inflammatory regulators in the development of PE. Various investigations have delved into the pathophysiological roles of cytokines, including tumor necrosis factor alpha (TNF-α), interleukin-6 (IL-6), interleukin-8 (IL-8), interleukin-17 (IL-17), interleukin-18 (IL-18), interferon gamma (IFN-γ), interleukin-4 (IL-4), and interleukin-10 (IL-10) in the progression of PE (Lau et al., 2013; Yang et al., 2014; Guan et al., 2023). However, due to the presence of residual confounders, the conclusive establishment of a causal relationship between cytokines and PE remains elusive.

Mendelian randomization (MR) studies stand out as a distinctive and potent statistical approach to investigate causality between exposures (e.g., circulating cytokines) and outcomes (e.g., preeclampsia) of interest (Smith and Ebrahim, 2003; Hemani et al., 2018). This method uses genetic instruments as unconfounded proxies for exposures, which can avoid residual confounding and reverse causality that is commonly present in conventional observational studies (Burgess et al., 2013). In the absence of randomized clinical trials (RCTs), MR design is an important strategy for causal inference as genetic variants are randomly assorted at meiosis, in which the procedure mimics an RCT (Lawlor et al., 2008). Furthermore, a two-sample MR design using summary statistics from a genome-wide association study (GWAS) greatly increases the statistical power of causality inference (Pierce and Burgess, 2013). Two-sample MR analysis allows researchers to evaluate the relationship between instrument exposure and instrument results in two separate population samples, thereby enhancing the applicability and effectiveness (Hartwig et al., 2016).

Prior MR studies have investigated the causal influence of systemic inflammatory regulators on various diseases. Yeung et al. discovered that the considered systemic inflammatory regulators did not impact the risk of Alzheimer’s disease (AD). In contrast, specific cytokines such as interleukin-2 (IL-2), IFN-γ, TNF-α, and IL-18 might be downstream effects of AD (Yeung and Schooling, 2021). Song et al. (2022) demonstrated that elevated levels of IL-18 were correlated with a reduced risk of acute myeloid leukemia, while IL-17 was associated with the risk of stomach cancer. Wang et al. proposed that heightened levels of monocyte-specific chemokine-3 (MCP3), vascular endothelial growth factor (VEGF), IL-10, and IL-7 were linked to an increased risk of multiple myeloma (MM), whereas lower levels of tumor necrosis factor beta (TNF-β) were strongly associated with an elevated risk of MM (Wang et al., 2022).

Nevertheless, the bidirectional association between systemic inflammatory regulators and PE has not been explored to date. In this study, we employed a two-sample MR analysis to investigate the potential causal relationship between systemic inflammatory regulators and PE and *vice versa*.

## Materials and methods

### Study design

The summary data for the GWAS on systemic inflammatory regulators and PE were obtained from previously published studies (refer to Supplementary Table S1), obviating the need for additional ethical clearance. This study adheres to the STROBE-MR guidelines, as outlined by Skrivankova et al. (2021). The fulfillment of the three principal criteria is imperative, as delineated by VanderWeele et al. (2014) (Ives et al., 2020): instrumental variables (IVs) exhibit a robust association with the exposure (Brown et al., 2018); IVs are independent of any unidentified confounders existing between the exposure and outcome (Poon et al., 2019); and IVs exclusively influence the outcome through their impact on the exposure.

### Data sources and genetic associations.

Summary-level data for 41 systemic inflammatory regulators were acquired in this study from a GWAS, encompassing 8,293 individuals of Finnish descent from 3 distinct cohort studies: the Cardiovascular Risk in Young Finns Study, the FINRISK 1997 study, and the FINRISK 2002 study (Ahola-Olli et al., 2017). To ensure robust genetic associations, adjustments were made for age, sex, body mass index, and the top 10 genetic principal components.

The genetic association data pertaining to PE were sourced from a meta-analysis that included data from the UK Biobank, FinnGen, and BioBank. This meta-analysis involved 2,355 cases and 264,887 controls of European ancestry, as reported by Sakaue et al. (2021). Notably, this study represents a multi-country GWAS meta-analysis with minimal overlap with the GWAS on inflammatory regulators. Additionally, all single-nucleotide polymorphisms (SNPs) were derived from the analysis of European populations, thereby mitigating potential biases associated with population differences.

### Selection of genetic instrumental variables

To mitigate the risk of false-positive IVs, we opted for statistically significant criteria (*p* < 5 × 10^−6^, linkage disequilibrium (LD) *r* ^2^ < 0.001, and window size = 10,000 kb) in the summary data for systemic inflammatory regulators, as recommended by Rayes et al. (2023). Following the harmonization of the selected SNPs with those associated with PE, we identified a total of 41 circulating cytokines. To ensure the robustness of the instrumental variable, we verified that the *F*-statistics exceeded 10, adhering to the stringent mathematical formula: *F* = β^2^_exposure/SE^2^_exposure. The details of the 41 systemic inflammatory regulators chosen are provided in Supplementary Table S2.

For selecting IVs of PE, LD (*r* ^2^ < 0.001) and proxies were tested for the 14 SNPs estimated to be correlated to PE at the genome-wide significance level (*p* < 5 × 10^−6^). Eventually, after harmonizing the exposure–outcome datasets, nine SNPs in total were included to construct the genetic IVs for PE (Supplementary Table S3).

### Statistics

We systematically validated potential causal relationships between systemic inflammatory regulators and PE through a robust methodology. Primary MR analyses were conducted using the inverse-variance weighted (IVW) approach with random effects, utilizing genetic data to estimate causal effects between exposures and outcomes. Odds ratios (ORs) and 95% confidence intervals (CIs) were reported, with statistical significance set at *p* < 0.05.

To enhance the reliability of our MR results, we implemented various heterogeneity and sensitivity assessments. Sensitivity analysis involved the utilization of the MR–Egger, weighted median, weighted mode, and simple mode techniques, as detailed by Xu et al. (2017). The MR–Egger method, employed to assess bias due to gene pleiotropic effects, demonstrated potential susceptibility and lower precision to a weak instrumental variable bias than other methods such as IVW and weighted median. The MR–Egger intercept was employed to identify horizontal pleiotropy, with statistical significance set at *p* < 0.05.

To further scrutinize pleiotropy and correct horizontal pleiotropy through outlier removal with potential pleiotropy, we employed MR Pleiotropy RESidual Sum and Outlier (MR-PRESSO) as proposed by Ong and MacGregor (2019) and Verbanck et al. (2018). Cochran’s Q test was utilized to evaluate heterogeneity and horizontal pleiotropy. The analysis of Cochran’s Q test results and funnel plots served as indices to estimate individual SNP heterogeneity, in accordance with the approach outlined by Bowden et al. (2018).

Additionally, we visually presented the results through scatter plots, forest plots, and leave-one-out plots, following the methodology described by Hemani et al. (2018). This comprehensive validation process strengthens the robustness and credibility of our findings regarding the causal relationships between systemic inflammatory regulators and PE. To account for multiple testing, we applied the Bonferroni method, which requires us to calculate associations with *p*-values below 0.0012 (0.05/41) as strong evidence of associations. The results with *p*-values ranging from 0.0012 to 0.05 were regarded as suggestive associations (Shi et al., 2023). Statistical analyses were performed and visualized using the R program (version 4.3.0) with the two-sample MR package (version 0.5.6) and MR-PRESSO (version 1.0) (Hemani et al., 2018).

## Results

### The causal effects of systemic inflammatory regulators on preeclampsia

The MR analysis uncovered a significant association between specific systemic inflammatory regulators—TNF-α, stem cell growth factor beta (SCGF-β), IL-9, and IL-5—and the risk of PE. All SNPs exhibited *F*-statistics greater than 10 (range 20.35–99.619; see Supplementary Table S2). Fourteen IVs were identified for SCGF-β, while IL-9, IL-5, and TNF-α had six, five, and five IVs, respectively. The IVW method demonstrated a significant difference (*p* < 0.05), and consistent directional effects were observed across five methods, namely, IVW, MR–Egger, weighted median, weighted mode, and simple mode (refer to Supplementary Table S4).

The IVW analysis revealed a negative association between SCGF-β (OR = 0.89, 95% CI: 0.80–0.99, *p* = 0.027) and IL-5 (OR = 0.80, 95% CI: 0.65–0.98, *p* = 0.030), indicating a decreased risk of PE. Although increased levels of interferon gamma-induced protein 10 (IP10) were associated with a reduced risk of PE, the IVW test results were inconclusive (*p* > 0.05), prompting the use of the MR–Egger method (OR = 0.66, 95% CI = 0.49–0.89, *p* = 0.026). The MR-Egger intercept did not indicate potential horizontal pleiotropy for SCGF-β and IL-5 (*p* = 0.968; *p* = 0.732, respectively), while IP10 exhibited pleiotropy (*p* = 0.033) (refer to Supplementary Tables S5, S6). Given that horizontal pleiotropy violates MR assumptions, the analysis involving IP10 was considered unreliable. Additionally, Cochran’s Q values based on the IVW tests showed no significant heterogeneity for SCGF-β and IL-5 (all *p* > 0.05; Supplementary Table S7).

Similarly, the IVW method revealed that elevated circulating levels of TNF-α and IL-9 were causally related to an increased risk of PE (OR = 1.32, 95% CI = 1.09–1.60, *p* = 0.004 and OR = 1.28, 95% CI: 1.02–1.62, *p* = 0.033, respectively). The MR–Egger intercept did not detect potential horizontal pleiotropy for TNF-α and IL-9 (*p* = 0.620 and *p* = 0.588, respectively). Cochran’s Q values based on the IVW tests indicated no significant heterogeneity for SCGF-β and IL-5 (all *p* > 0.05).

**FIGURE 1:**
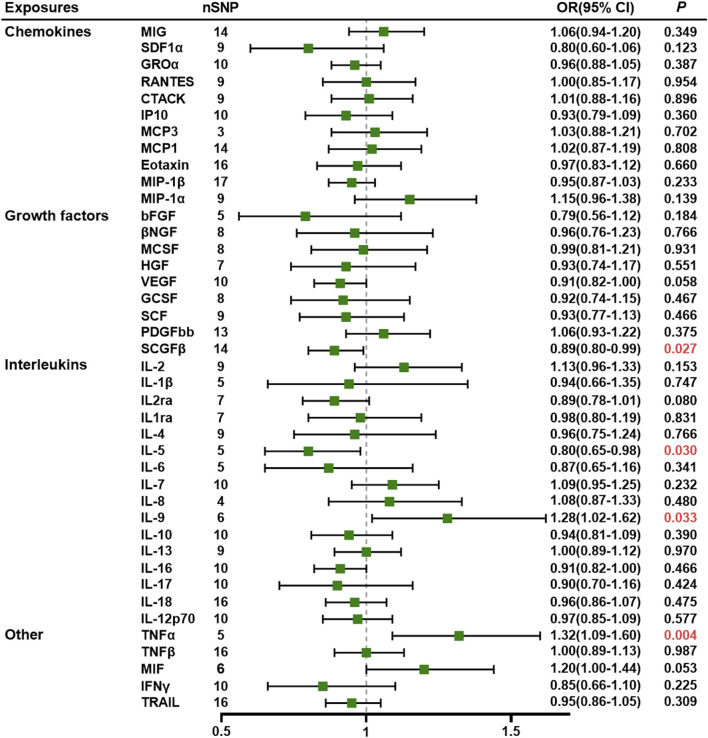
Association of systemic inflammatory regulators with preeclampsia using Mendelian randomization (MR) (with SNPs) reaching *p* < 5 × 10^−6^).The inverse-variance weighted (IVW) method was used as the primary method for MR analysis. OR, odds ratio; CI, confidence interval; CTACK, cutaneous T-cell-attracting chemokine; GCSF, granulocyte colony-stimulating factor; GROα, growth-regulated oncogene-α; HGF, hepatocyte growth factor; βNGF, beta nerve growth factor; bFGF, basic fibroblast growth factor; IFN-γ, interferon gamma; IL, interleukin; IP10, interferon-gamma-induced protein 10; MCP1, monocyte chemotactic protein-1; MCP3, monocyte chemotactic protein-3; MCSF, macrophage colony-stimulating factor; MIF, macrophage migration inhibitory factor; MIG, monokine induced by interferon gamma; MIP-1α, macrophage inflammatory protein-1α; MIP-1β, macrophage inflammatory protein-1β; PDGF-BB, platelet-derived growth factor-BB; SCGF-β, stem cell growth factor beta; SDF-1α, stromal cell-derived factor-1α; SNPs, single-nucleotide polymorphisms; TNF, tumor necrosis factor; RANTES, regulated on activation, normal T cell expressed and secreted; SCF, stem cell factor; TRAIL, TNF-related apoptosis-inducing ligand; VEGF, vascular endothelial growth factor.

The MR results, along with heterogeneity analysis, pleiotropy analysis, and sensitivity analysis results, are summarized in Supplementary Tables S5–S7. No SNP significantly influenced the overall effect of cytokines on PE in the IVW leave-one-out sensitivity analysis. Supplementary Figures S1–S5 display scatter plots, funnel plots, forest plots, and leave-one-out plots, while Figure 1 presents the forest plots of the obtained results.

Furthermore, other systemic inflammatory regulators, including cutaneous T-cell-attracting chemokine, beta nerve growth factor, VEGF, macrophage migration inhibitory factor (MIF), TNF-related apoptosis-inducing ligand, TNF-β, MCP3, stromal cell-derived factor-1 alpha, stem cell factor, interleukin-12p70, interleukin-16, platelet-derived growth factor-BB, growth-regulated protein alpha, hepatocyte growth factor, interleukin-1 receptor antagonist, monocyte chemoattractant protein-1, macrophage inflammatory protein 1b, and IL-18, did not exhibit significant associations with PE in any of the analyses.

### The causal effects of preeclampsia on systemic inflammatory regulators

**FIGURE 2:**
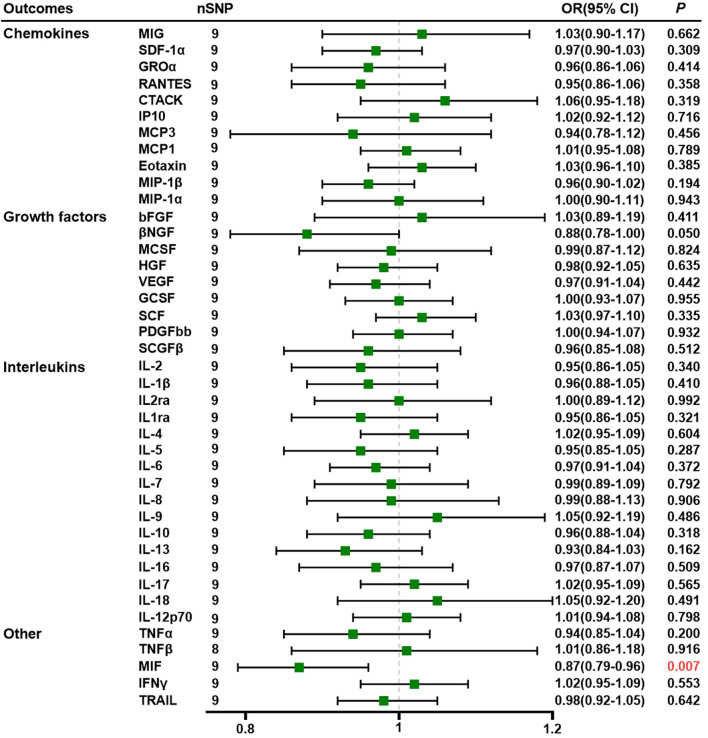
Association of preeclampsia with systemic inflammatory regulators using Mendelian randomization (with SNPs reaching *p* < 5 × 10^−6^). The IVW method was used as the primary method for MR analysis. OR, odds ratio; CI, confidence interval; CTACK, cutaneous T-cell-attracting chemokine; GCSF, granulocyte colony-stimulating factor; GROα, growth-regulated oncogene-a; HGF, hepatocyte growth factor; βNGF, beta nerve growth factor; bFGF, basic fibroblast growth factor; IFN-γ, interferon gamma; IL, interleukin; IP10, interferon-gamma-induced protein 10; MCP1, monocyte chemotactic protein-1; MCP3, monocyte chemotactic protein-3; MCSF, macrophage colony-stimulating factor; MIF, macrophage migration inhibitory factor; MIG, monokine induced by interferon gamma; MIP-1α, macrophage inflammatory protein-1α; MIP-1β, macrophage inflammatory protein-1β; PDGF-BB, platelet-derived growth factor BB; SCGF-β, stem cell growth factor beta; SDF-1α, stromal cell-derived factor-1α; SNPs, single-nucleotide polymorphisms; TNF, tumor necrosis factor; RANTES, regulated on activation, normal T cell expressed and secreted; SCF, stem cell factor; TRAIL, TNF-related apoptosis-inducing ligand; VEGF, vascular endothelial growth factor.

Using nine SNPs as IVs for PE, we demonstrated that genetically predicted PE is negatively associated with MIF levels through the IVW method (OR = 0.87, 95% CI = 0.79–0.96, *p* = 0.007) (Supplementary Tables S8). All SNPs we selected had *F*-statistics greater than 10 (mean, 22.83; range, 20.97–27.69). The MR–Egger intercept did not detect potential horizontal pleiotropy (*p* = 0.705; Supplementary Tables S9). Cochran’s Q values based on the IVW tests showed that there was no obvious heterogeneity (*p* = 0.434). Detailed data are given in Supplementary Tables S10, S11. Figure 2 shows the forest plots of the above results. Scatter plots, funnel plots, forest plots, and leave-one-out plots are listed in Supplementary Tables S5.

## Discussion

The goal of this extensive two-sample bidirectional MR investigation was to examine the causal relationship between systemic inflammatory regulators and PE by analyzing the large-scale summary GWAS data that are now available. It was discovered that none of these passed statistical significance after correction for multiple testing, although several suggestive results demonstrated biological plausibility. A higher risk of PE is linked to higher levels of TNF-α and IL-9 and lower levels of SCGF-β and IL-5. Genetically determined PE may also be a factor in a lower MIF level. This effect was further corroborated in a sensitivity analysis and was directionally consistent across various MR analyses.

There have been contradictory findings from earlier systematic reviews and meta-analyses of observational cohorts. They agreed with our results of TNF-α, IL-9, and IL-5 (6, 28, 29). Nonetheless, the contradictory findings were taken from observational research and combined. Reverse caution and confounding variables may skew the true causal links. Using a two-sample bidirectional MR analysis, we identified the inflammatory regulators of PE that are downstream (MIF) and upstream (TNF-α, IL-9, IL-5, and SCGF-β).

TNF-α is a multifunctional T-helper type 1 (Th1) cytokine and one of the most important inflammatory cytokines. TNF-α can induce structural and functional alterations in endothelial cells, enhancing the formation and release of endothelin and decreasing acetylcholine-induced vasodilating factors, such as nitric oxide (LaMarca et al., 2005). A meta-analysis revealed that the maternal level of TNF-α was significantly higher in PE than that in the control group (mean difference = 8.11 pg/mL, 95% CI = 5.87–10.34 pg/mL) (Lau et al., 2013; Spence et al., 2021). A report, therefore, proposed the use of etanercept for gestational hypertension (Fu et al., 2019). Progesterone directly suppressed TNF-α-stimulated endothelin (ET)-1 and attenuated TNF-α-induced hypertension, possibly via the suppression of the renal ET-1 system (Keiser et al., 2009). Furthermore, dydrogesterone can cause a significantly reduced secretion of the Th1 cytokine TNF-α (Raghupathy, 2013). Anti-TNF-α appears to be a potential therapeutic approach for PE. The most common adverse pregnancy outcomes following the use of TNF-α inhibitors (e.g., etanercept) are intrauterine growth restriction, spontaneous abortion, and preterm birth (Dai et al., 2022). So, how to balance the advantages and disadvantages of the drugs and which medications should be chosen at different trimesters of pregnancy have no consensus in clinical studies. SCGF-β is a hematopoietic growth factor that exerts its cellular activity at the early stage of hematopoiesis (Sukowati et al., 2018). No research focusing on the link between SCGF-β and PE has been identified thus far. Further investigation may be necessary to explore the potential use of SCGF-β as a biomarker for PE.

Additionally, IL-9, characterized as a T-cell growth factor and a member of the common γ-chain receptor cytokine family, originates from Th2 cells, Th9 cells, Th17 cells, Treg cells, mast cells, and natural killer cells (Noelle and Nowak, 2010). IL-9 is implicated in maintaining pregnancy and laboring activity (Habbeddine et al., 2014; Sun et al., 2021). It has the potential to enhance the suppressive functions of Treg cells and the production of TGF-β by antigen-presenting cells, thereby preventing maternal immune activation against the fetus (Noelle and Nowak, 2010). IL-9 accelerates the *in vitro* formation of trophoblastic capillary tubes, and first-trimester serum IL-9 levels differ significantly between preeclamptic and normotensive pregnant women (Liu et al., 2023). In contrast to our findings, Sun et al. suggested that reduced IL-9 activity might lead to poorer angiogenesis and inadequate remodeling of the maternal uterine spiral arteries, potentially contributing to PE (Sun et al., 2020).

Regarding IL-5, previous studies have reported that IL-5, a Th2 cytokine, stimulates the proliferation, migration, and tube formation of human umbilical vein endothelial cells by activating the endothelial nitric oxide synthase pathway. Lower levels of IL-5 were observed across preeclamptic women in early pregnancy than that in normotensive women (Tangeras et al., 2015; Park et al., 2017). This observational result aligns with our genetically based assumption. However, more clinical and animal studies are needed to elucidate the correlation between IL-5 and PE.

MIF promotes trophoblast migration, invasion, and remodeling spiral artery processes (Jovanovic Krivokuca et al., 2015; Vilotic et al., 2019). Abundant studies have described that placental soluble fms-like tyrosine kinase 1 (sFlt1) levels increase significantly in PE compared to normotensive pregnancy, while placental MIF positively correlates with sFlt1 expression (Yong et al., 2023). Reports on the comparison of serum MIF content between preeclamptic and normotensive pregnancies are controversial (Todros et al., 2005; Hristoskova et al., 2006; Cardaropoli et al., 2014). No evidence shows that MIF is a downstream inflammatory regulator of PE so far. MIF measurement in PE might be of value, but additional research is needed to establish the reference range of serum MIF.

This study has several strengths. To the best of our knowledge, this is the first and most comprehensive MR study exploring the bidirectional relationship between 41 systemic inflammatory regulators and PE. Most studies investigating the link between systemic inflammatory regulators and PE relied on animal experiments and cross-sectional studies, precluding the ability to identify causality. We took the advantage of MR analysis to avoid reverse causal relations and minimize residual confounders. Furthermore, we used the latest version of systemic inflammatory regulators to conduct this MR study, making it the most up-to-date and comprehensive summary data in evaluating the causal relationship between systemic inflammatory regulators and PE. We restricted our study to women of European ancestry to avoid racial heterogeneity. Our study mainly focuses on both upstream and downstream circulating biomarkers that could depict the whole clinical picture in managing PE.

Nevertheless, there were some limitations. First, the large-scale GWAS data were summative, precluding further analysis at the individual-level data. The summary data for systemic inflammatory regulators encompassed both females and males, with gender differences excluding genetic variants on sex chromosomes (Pierce and Burgess, 2013). It is important to note that this two-sample Mendelian randomization analysis was confined to the European population, and caution should be exercised in generalizing the conclusions to other ethnic groups due to genetic variations among different races (Tan et al., 2021). To comprehensively explore potential causality across races, efforts should be made to uncover more genetic information from diverse ethnic backgrounds.

Furthermore, it is worth acknowledging that MR may not be the optimal approach, considering that the exposure of interest can vary throughout life. However, the genetic instruments used in the analysis provide independent evidence from environmental or lifestyle factors, adding relevance to the study of PE.

In conclusion, we applied the bidirectional two-sample MR study to evaluate the causal effects of systemic inflammatory regulators on PE or *vice versa*. We then identified and validate the causal effect of IL-5, IL-9, TNF-α, and SCGF-β on PE. Conversely, MIF is a downstream inflammatory regulator of PE. Further efforts should be made to explore the precise contribution of systemic inflammatory regulators to the pathophysiology of PE. This will improve the management of PE in terms of early prediction, prevention, and treatment.

## Data Availability

The original contributions presented in the study are included in the article/Supplementary Material; further inquiries can be directed to the corresponding authors.

## References

[B1] Ahola-Olli A. V. Wurtz P. Havulinna A. S. Aalto K. Pitkanen N. Lehtimaki T. (2017). Genome-wide association study identifies 27 loci influencing concentrations of circulating cytokines and growth factors. Am. J. Hum. Genet. 100 (1), 40–50. 10.1016/j.ajhg.2016.11.007 27989323 PMC5223028

[B2] Bowden J. Spiller W. Del Greco M. F. Sheehan N. Thompson J. Minelli C. (2018). Improving the visualization, interpretation and analysis of two-sample summary data Mendelian randomization via the Radial plot and Radial regression. Int. J. Epidemiol. 47 (4), 1264–1278. 10.1093/ije/dyy101 29961852 PMC6124632

[B3] Brown M. A. Magee L. A. Kenny L. C. Karumanchi S. A. McCarthy F. P. Saito S. (2018). The hypertensive disorders of pregnancy: ISSHP classification, diagnosis & management recommendations for international practice. Pregnancy Hypertens. 13, 291–310. 10.1016/j.preghy.2018.05.004 29803330

[B4] Burgess S. Butterworth A. Thompson S. G. (2013). Mendelian randomization analysis with multiple genetic variants using summarized data. Genet. Epidemiol. 37 (7), 658–665. 10.1002/gepi.21758 24114802 PMC4377079

[B5] Cardaropoli S. Ietta F. Romagnoli R. Rolfo A. Paulesu L. Todros T. (2014). Lower macrophage migration inhibitory factor concentrations in maternal serum before pre-eclampsia onset. J. Interferon Cytokine Res. 34 (7), 537–542. 10.1089/jir.2013.0057 24606610

[B6] Dai F. F. Hu M. Zhang Y. W. Zhu R. H. Chen L. P. Li Z. D. (2022). TNF-*α*/anti-TNF-*α* drugs and its effect on pregnancy outcomes. Expert Rev. Mol. Med. 24, e26. 10.1017/erm.2022.18 35687009 PMC9884758

[B7] Fu J. Li L. Qi L. Zhao L. (2019). A randomized controlled trial of etanercept in the treatment of refractory recurrent spontaneous abortion with innate immune disorders. Taiwan J. Obstet. Gynecol. 58 (5), 621–625. 10.1016/j.tjog.2019.07.007 31542082

[B8] Guan X. Fu Y. Liu Y. Cui M. Zhang C. Zhang Q. (2023). The role of inflammatory biomarkers in the development and progression of pre-eclampsia: a systematic review and meta-analysis. Front. Immunol. 14, 1156039. 10.3389/fimmu.2023.1156039 37325643 PMC10266420

[B9] Habbeddine M. Verbeke P. Karaz S. Bobe P. Kanellopoulos-Langevin C. (2014). Leukocyte population dynamics and detection of IL-9 as a major cytokine at the mouse fetal-maternal interface. PLoS One 9 (9), e107267. 10.1371/journal.pone.0107267 25259859 PMC4178026

[B10] Hartwig F. P. Davies N. M. Hemani G. Davey Smith G. (2016). Two-sample Mendelian randomization: avoiding the downsides of a powerful, widely applicable but potentially fallible technique. Int. J. Epidemiol. 45 (6), 1717–1726. 10.1093/ije/dyx028 28338968 PMC5722032

[B11] Hemani G. Zheng J. Elsworth B. Wade K. H. Haberland V. Baird D. (2018). The MR-Base platform supports systematic causal inference across the human phenome. Elife 7, e34408. 10.7554/eLife.34408 29846171 PMC5976434

[B12] Hristoskova S. Holzgreve W. Zhong X. Y. Hahn S. (2006). Macrophage migration inhibition factor is elevated in pregnancy, but not to a greater extent in preeclampsia. Arch. Gynecol. Obstet. 274 (1), 25–28. 10.1007/s00404-005-0109-8 16369812

[B13] Ives C. W. Sinkey R. Rajapreyar I. Tita A. T. N. Oparil S. (2020). Preeclampsia-pathophysiology and clinical presentations: JACC state-of-the-art review. J. Am. Coll. Cardiol. 76 (14), 1690–1702. 10.1016/j.jacc.2020.08.014 33004135

[B14] Jonsson Y. Matthiesen L. Berg G. Ernerudh J. Nieminen K. Ekerfelt C. (2005). Indications of an altered immune balance in preeclampsia: a decrease in *in vitro* secretion of IL-5 and IL-10 from blood mononuclear cells and in blood basophil counts compared with normal pregnancy. J. Reprod. Immunol. 66 (1), 69–84. 10.1016/j.jri.2005.02.002 15949563

[B15] Jovanovic Krivokuca M. Stefanoska I. Abu Rabi T. Al-Abed Y. Stosic-Grujicic S. Vicovac L. (2015). Pharmacological inhibition of MIF interferes with trophoblast cell migration and invasiveness. Placenta 36 (2), 150–159. 10.1016/j.placenta.2014.12.003 25530499

[B16] Keiser S. D. Veillon E. W. Parrish M. R. Bennett W. Cockrell K. Fournier L. (2009). Effects of 17-hydroxyprogesterone on tumor necrosis factor-alpha-induced hypertension during pregnancy. Am. J. Hypertens. 22 (10), 1120–1125. 10.1038/ajh.2009.149 19745821 PMC2810643

[B17] Kvalvik L. G. Wilcox A. J. Skjaerven R. Ostbye T. Harmon Q. E. (2020). Term complications and subsequent risk of preterm birth: registry based study. BMJ 369, m1007. 10.1136/bmj.m1007 32349968 PMC7188013

[B18] LaMarca B. B. Cockrell K. Sullivan E. Bennett W. Granger J. P. (2005). Role of endothelin in mediating tumor necrosis factor-induced hypertension in pregnant rats. Hypertension 46 (1), 82–86. 10.1161/01.HYP.0000169152.59854.36 15928030

[B19] Lau S. Y. Guild S. J. Barrett C. J. Chen Q. McCowan L. Jordan V. (2013). Tumor necrosis factor-alpha, interleukin-6, and interleukin-10 levels are altered in preeclampsia: a systematic review and meta-analysis. Am. J. Reprod. Immunol. 70 (5), 412–427. 10.1111/aji.12138 23790133

[B20] Lawlor D. A. Harbord R. M. Sterne J. A. Timpson N. Davey Smith G. (2008). Mendelian randomization: using genes as instruments for making causal inferences in epidemiology. Stat. Med. 27 (8), 1133–1163. 10.1002/sim.3034 17886233

[B21] Liu M. Niu Y. Ma K. Leung P. C. K. Chen Z. J. Wei D. (2023). Identification of novel first-trimester serum biomarkers for early prediction of preeclampsia. J. Transl. Med. 21 (1), 634. 10.1186/s12967-023-04472-1 37718445 PMC10506221

[B22] Noelle R. J. Nowak E. C. (2010). Cellular sources and immune functions of interleukin-9. Nat. Rev. Immunol. 10 (10), 683–687. 10.1038/nri2848 20847745 PMC3828627

[B23] Ong J. S. MacGregor S. (2019). Implementing MR-PRESSO and GCTA-GSMR for pleiotropy assessment in Mendelian randomization studies from a practitioner's perspective. Genet. Epidemiol. 43 (6), 609–616. 10.1002/gepi.22207 31045282 PMC6767464

[B24] Park S. L. Chung T. W. Kim S. Hwang B. Kim J. M. Lee H. M. (2017). HSP70-1 is required for interleukin-5-induced angiogenic responses through eNOS pathway. Sci. Rep. 7, 44687. 10.1038/srep44687 28317868 PMC5357797

[B25] Pierce B. L. Burgess S. (2013). Efficient design for Mendelian randomization studies: subsample and 2-sample instrumental variable estimators. Am. J. Epidemiol. 178 (7), 1177–1184. 10.1093/aje/kwt084 23863760 PMC3783091

[B26] Pittara T. Vyrides A. Lamnisos D. Giannakou K. (2021). Pre-eclampsia and long-term health outcomes for mother and infant: an umbrella review. BJOG 128 (9), 1421–1430. 10.1111/1471-0528.16683 33638891

[B27] Poon L. C. Shennan A. Hyett J. A. Kapur A. Hadar E. Divakar H. (2019). The International Federation of Gynecology and Obstetrics (FIGO) initiative on pre-eclampsia: a pragmatic guide for first-trimester screening and prevention. Int. J. Gynaecol. Obstet. 145 (1), 1–33. 10.1002/ijgo.12802 PMC6944283 31111484

[B28] Raghupathy R. (2013). Cytokines as key players in the pathophysiology of preeclampsia. Med. Princ. Pract. 22, 8–19. 10.1159/000354200 23949305 PMC5586811

[B29] Rayes B. Ardissino M. Slob E. A. W. Patel K. H. K. Girling J. Ng F. S. (2023). Association of hypertensive disorders of pregnancy with future cardiovascular disease. JAMA Netw. Open 6 (2), e230034. 10.1001/jamanetworkopen.2023.0034 36800181 PMC9938428

[B30] Sakaue S. Kanai M. Tanigawa Y. Karjalainen J. Kurki M. Koshiba S. (2021). A cross-population atlas of genetic associations for 220 human phenotypes. Nat. Genet. 53 (10), 1415–1424. 10.1038/s41588-021-00931-x 34594039 PMC12208603

[B31] Shi Q. Wang Q. Wang Z. Lu J. Wang R. (2023). Systemic inflammatory regulators and proliferative diabetic retinopathy: a bidirectional Mendelian randomization study. Front. Immunol. 14, 1088778. 10.3389/fimmu.2023.1088778 36845092 PMC9950638

[B32] Skrivankova V. W. Richmond R. C. Woolf B. A. R. Yarmolinsky J. Davies N. M. Swanson S. A. (2021). Strengthening the reporting of observational studies in epidemiology using mendelian randomization: the STROBE-MR statement. JAMA 326 (16), 1614–1621. 10.1001/jama.2021.18236 34698778

[B33] Smith G. D. Ebrahim S. (2003). 'Mendelian randomization': can genetic epidemiology contribute to understanding environmental determinants of disease? Int. J. Epidemiol. 32 (1), 1–22. 10.1093/ije/dyg070 12689998

[B34] Song J. Li A. Qian Y. Liu B. Lv L. Ye D. (2022). Genetically predicted circulating levels of cytokines and the risk of cancer. Front. Immunol. 13, 886144. 10.3389/fimmu.2022.886144 35865545 PMC9294168

[B35] Spence T. Allsopp P. J. Yeates A. J. Mulhern M. S. Strain J. J. McSorley E. M. (2021). Maternal serum cytokine concentrations in healthy pregnancy and preeclampsia. J. Pregnancy 2021, 6649608. 10.1155/2021/6649608 33680514 PMC7925069

[B36] Sukowati C. H. C. Patti R. Pascut D. Ladju R. B. Tarchi P. Zanotta N. (2018). Serum stem cell growth factor beta for the prediction of therapy response in hepatocellular carcinoma. Biomed. Res. Int. 2018, 6435482. 10.1155/2018/6435482 30246025 PMC6136561

[B37] Sun Y. Liu S. Hu R. Zhou Q. Li X. (2020). Decreased placental IL9 and IL9R in preeclampsia impair trophoblast cell proliferation, invasion, and angiogenesis. Hypertens. Pregnancy 39 (3), 228–235. 10.1080/10641955.2020.1754852 32329646

[B38] Sun Y. Wu S. Zhou Q. Li X. (2021). Trophoblast-derived interleukin 9 mediates immune cell conversion and contributes to maternal-fetal tolerance. J. Reprod. Immunol. 148, 103379. 10.1016/j.jri.2021.103379 34534877

[B39] Tan J. S. Yan X. X. Wu Y. Gao X. Xu X. Q. Jiang X. (2021). Rare variants in MTHFR predispose to occurrence and recurrence of pulmonary embolism. Int. J. Cardiol. 331, 236–242. 10.1016/j.ijcard.2021.01.073 33571559

[B40] Tangeras L. H. Austdal M. Skrastad R. B. Salvesen K. A. Austgulen R. Bathen T. F. (2015). Distinct first trimester cytokine profiles for gestational hypertension and preeclampsia. Arterioscler. Thromb. Vasc. Biol. 35 (11), 2478–2485. 10.1161/ATVBAHA.115.305817 26404486

[B41] Todros T. Bontempo S. Piccoli E. Ietta F. Romagnoli R. Biolcati M. (2005). Increased levels of macrophage migration inhibitory factor (MIF) in preeclampsia. Eur. J. Obstet. Gynecol. Reprod. Biol. 123 (2), 162–166. 10.1016/j.ejogrb.2005.03.014 15894418

[B42] VanderWeele T. J. Tchetgen Tchetgen E. J. Cornelis M. Kraft P. (2014). Methodological challenges in mendelian randomization. Epidemiology 25 (3), 427–435. 10.1097/EDE.0000000000000081 24681576 PMC3981897

[B43] Verbanck M. Chen C. Y. Neale B. Do R. (2018). Detection of widespread horizontal pleiotropy in causal relationships inferred from Mendelian randomization between complex traits and diseases. Nat. Genet. 50 (5), 693–698. 10.1038/s41588-018-0099-7 29686387 PMC6083837

[B44] Vilotic A. Jovanovic Krivokuca M. Stefanoska I. Vrzic Petronijevic S. Petronijevic M. Vicovac L. (2019). Macrophage migration inhibitory factor is involved in endovascular trophoblast cell function *in vitro* . EXCLI J. 18, Doc1007. 10.17179/excli2019-1630 31762725 PMC6868918

[B45] Wang Q. Shi Q. Lu J. Wang Z. Hou J. (2022). Causal relationships between inflammatory factors and multiple myeloma: a bidirectional Mendelian randomization study. Int. J. Cancer 151 (10), 1750–1759. 10.1002/ijc.34214 35841389

[B46] Xu L. Borges M. C. Hemani G. Lawlor D. A. (2017). The role of glycaemic and lipid risk factors in mediating the effect of BMI on coronary heart disease: a two-step, two-sample Mendelian randomisation study. Diabetologia 60 (11), 2210–2220. 10.1007/s00125-017-4396-y 28889241 PMC6342872

[B47] Yang Y. Su X. Xu W. Zhou R. (2014). Interleukin-18 and interferon gamma levels in preeclampsia: a systematic review and meta-analysis. Am. J. Reprod. Immunol. 72 (5), 504–514. 10.1111/aji.12298 25060131

[B48] Yeung C. H. C. Schooling C. M. (2021). Systemic inflammatory regulators and risk of Alzheimer's disease: a bidirectional Mendelian-randomization study. Int. J. Epidemiol. 50 (3), 829–840. 10.1093/ije/dyaa241 33313759

[B49] Yong Q. Dijkstra K. L. van der Keur C. Bruijn J. A. Eikmans M. Baelde H. J. (2023). MIF increases sFLT1 expression in early uncomplicated pregnancy and preeclampsia. Int. J. Mol. Sci. 24 (12), 10050. 10.3390/ijms241210050 37373198 PMC10298851

