# Supplementary material for: Systemic inflammatory regulators and preeclampsia: a two-sample bidirectional Mendelian randomization study
Source: Front Genet. 2024 Mar 22;15:1359579. doi: 10.3389/fgene.2024.1359579 (PMC10995280; doi:10.3389/fgene.2024.1359579)
Supplement: Supplementary file 2 [file Table2.DOCX]

**
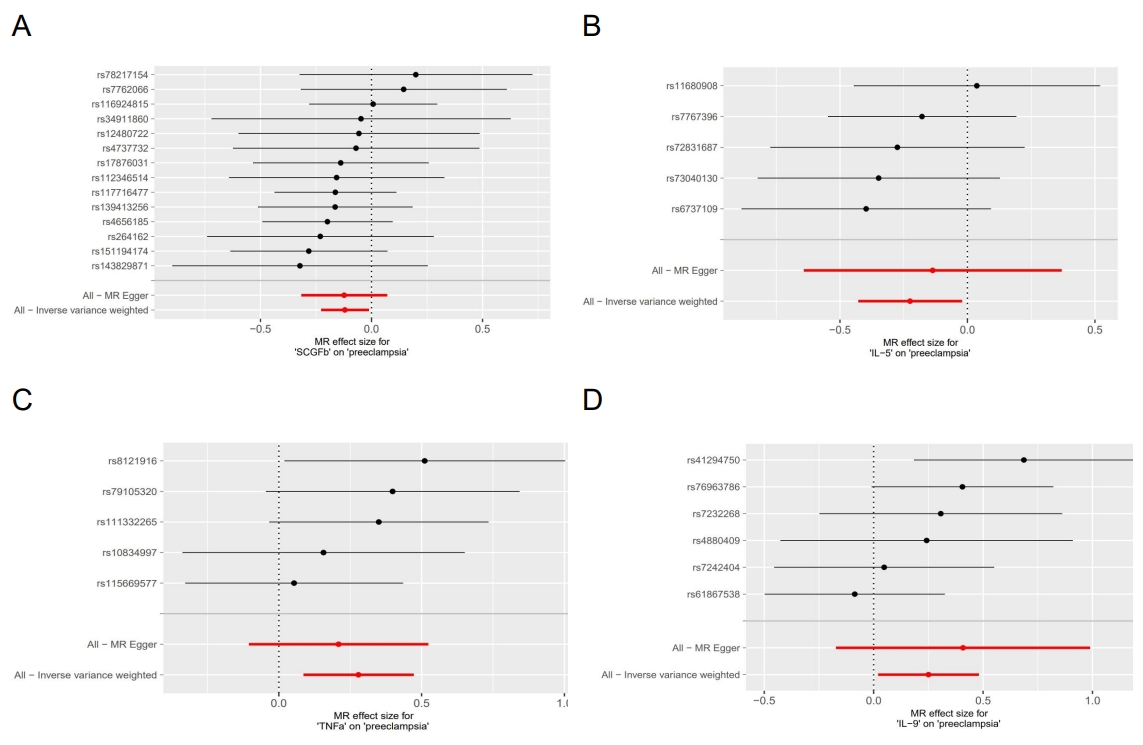
Figure S1.** Forest plots for the association of SCGFβ-, IL-5-, TNFα-, and IL-9-associated SNPs with risk of PE. (A) SCGFβ, (B) IL-5, (C) TNFα and (D) IL-9. SCGFβ, stem cell growth factor beta; IL-5, interleukin-5; TNFα, tumour necrosis factor α; IL-9, interleukin-9; PE, preeclampsia.


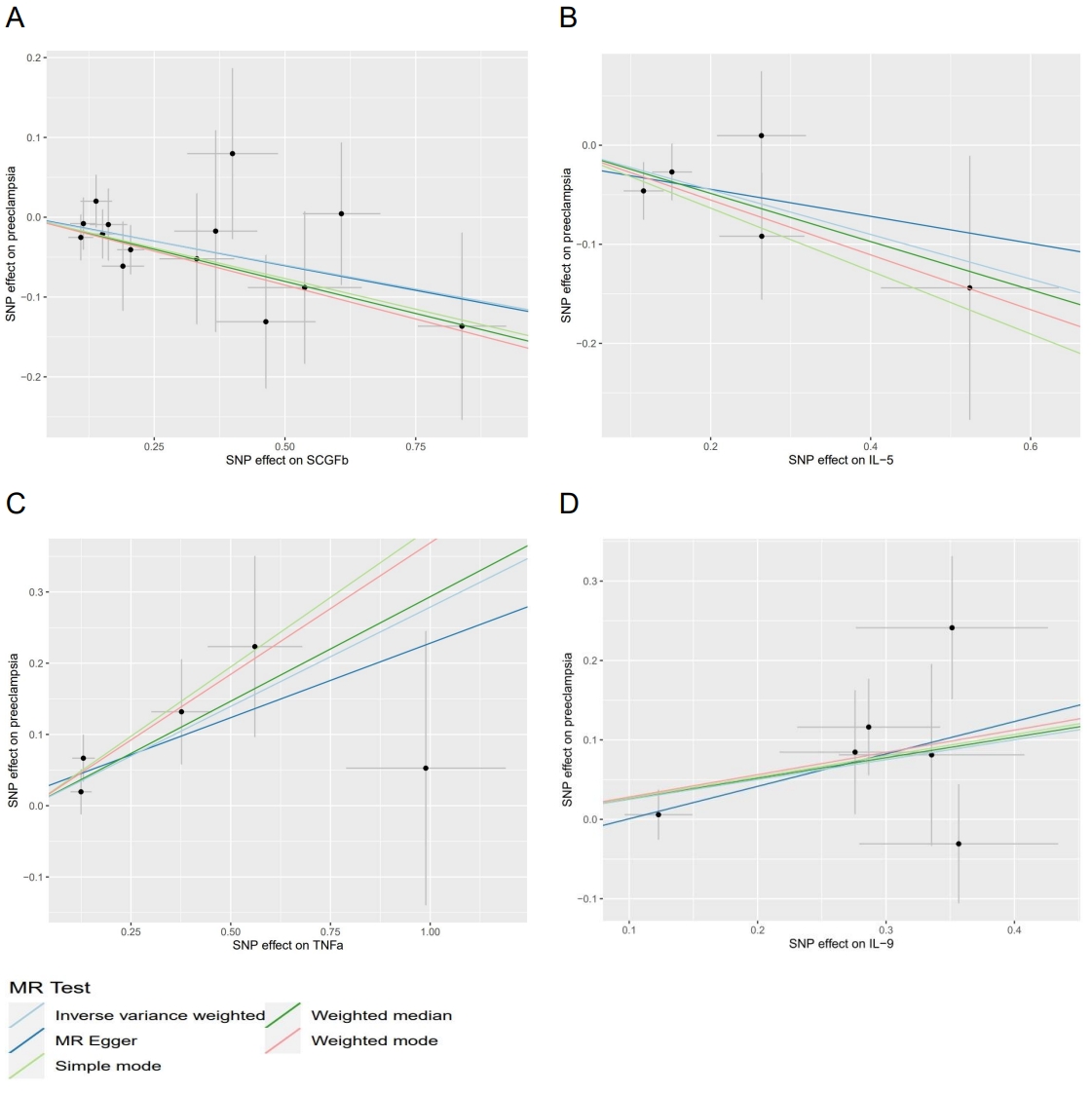


**Figure S2.** Scatter plots for the association of SCGFβ-, IL-5-, TNFα-, and IL-9-associated SNPs with risk of PE. (A) SCGFβ, (B) IL-5, (C) TNFα and (D) IL-9. SCGFβ, stem cell growth factor beta; IL-5, interleukin-5; TNFα, tumour necrosis factor α; IL-9, interleukin-9; PE, preeclampsia.


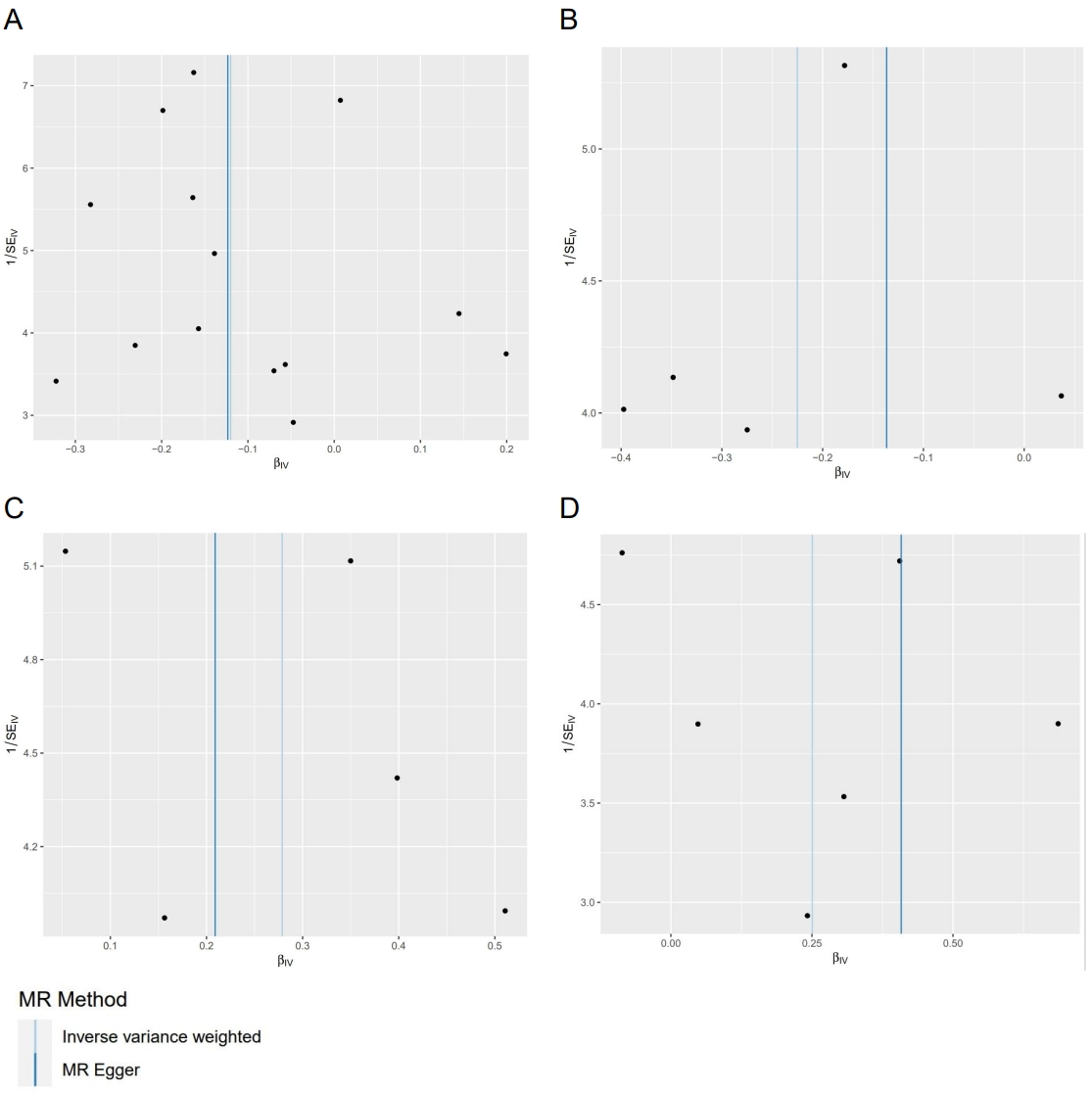


**Figure S3.** Funnel plots for the association of SCGFβ-, IL-5-, TNFα-, and IL-9-associated SNPs with risk of PE. (A) SCGFβ, (B) IL-5, (C) TNFα and (D) IL-9. SCGFβ, stem cell growth factor; IL-5, interleukin-5; TNFα, tumour necrosis factor α; IL-9, interleukin-9; PE, preeclampsia.

**
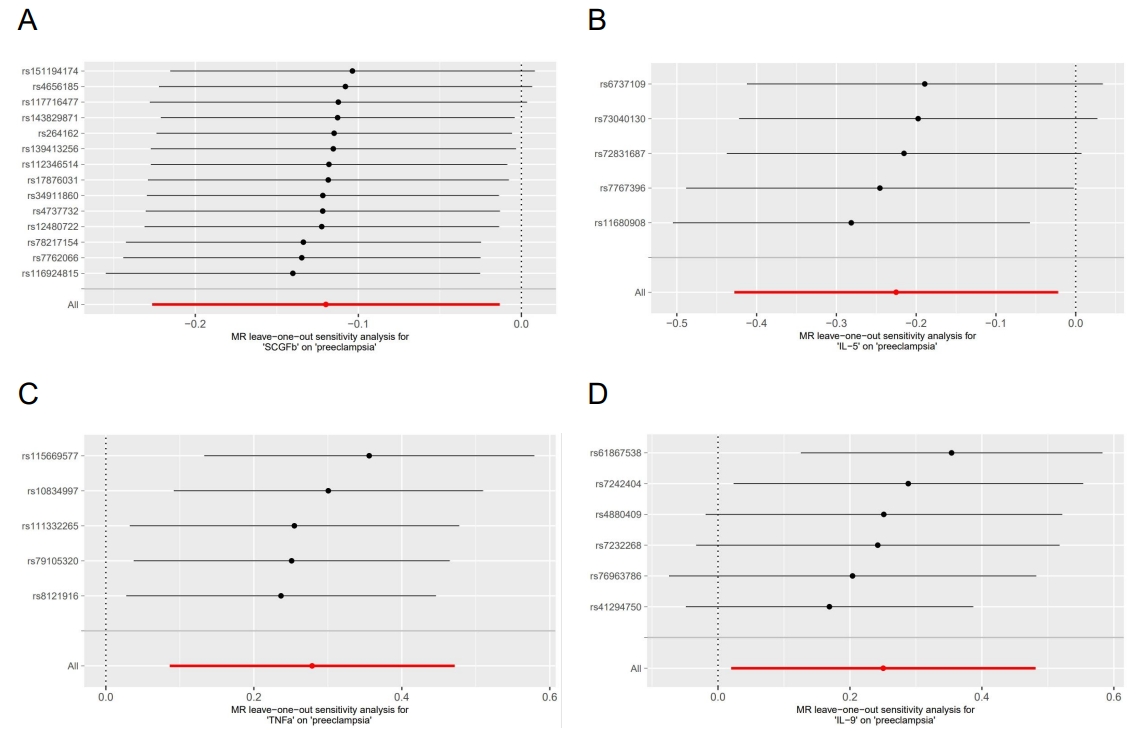
Figure S4.** Leave-one-out plots for the association of SCGFβ-, IL-5-, TNFα-, and IL-9-associated SNPs with risk of PE. (A) SCGFβ, (B) IL-5, (C) TNFα and (D) IL-9. SCGFβ, stem cell growth factor beta; IL-5, interleukin-5; TNFα, tumour necrosis factor α; IL-9, interleukin-9; PE, preeclampsia.


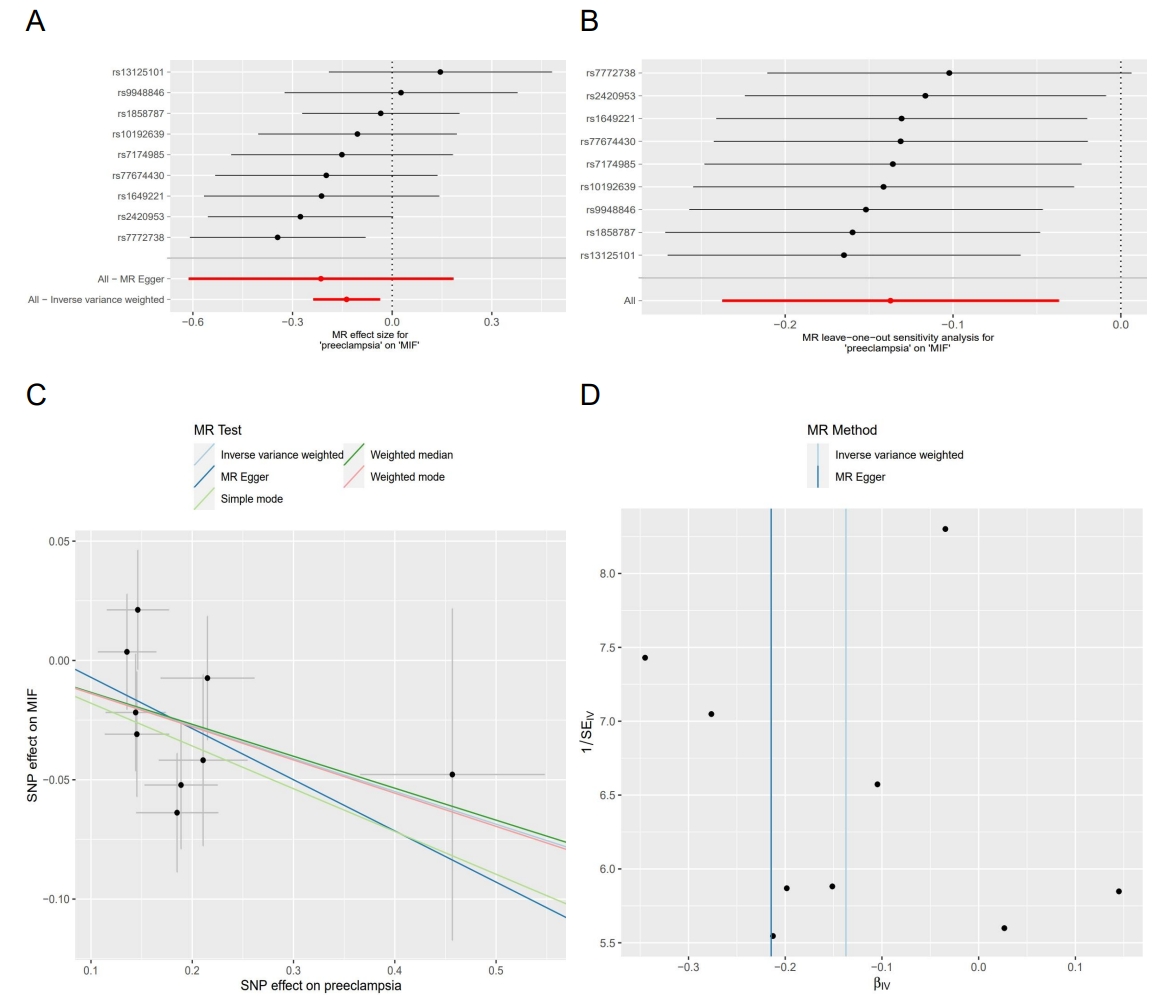


**Figure S5.** Summary plots for the association of PE associated SNPs with risk of MIF. (A) forest plots, (B) leave-one-out plots, (C) scatter plots and (D) funnel plots. MIF, macrophage migration inhibitory factor; PE, preeclampsia.
